# O.4.2-10 What can one minute of the day tell about physical capacity?

**DOI:** 10.1093/eurpub/ckad133.185

**Published:** 2023-09-11

**Authors:** Henri Vähä-Ypyä, Pauliina Husu, Kari Tokola, Harri Sievänen, Tommi Vasankari

**Affiliations:** UKK Institute, Finland; Henri.Vaha-Ypya@ukkinstituutti.fi; UKK Institute, Finland; Henri.Vaha-Ypya@ukkinstituutti.fi; UKK Institute, Finland; Henri.Vaha-Ypya@ukkinstituutti.fi; UKK Institute, Finland; Henri.Vaha-Ypya@ukkinstituutti.fi; UKK Institute, Finland; Henri.Vaha-Ypya@ukkinstituutti.fi

## Abstract

**Introduction:**

High cardiorespiratory fitness (CRF) allows individuals to perform daily activities and operate at higher intensity levels. This study investigates the association between the CRF and peak intensity of physical activity (PA) in absolute and relative terms. Absolute intensity measures the amount of energy expended per unit of time during an activity, while relative intensity reflects the proportion of an individual's maximal capacity.

**Methods:**

1952 adults (803 men, 1149 women) aged 20-69 years participated in the FinFit2017-study. Their CRF was predicted with 6 min walking test, and they wore the accelerometer for at least four days with a minimum of 24 h/day during the seven days measurement period. The participants were divided into CRF thirds by five age groups and sex. Acceleration data were analyzed in 6s epochs and intensity in MET (metabolic equivalent) values were calculated for each epoch. MET values were smoothed with a 1min exponential moving average and the highest daily value was recorded (peakMET). Analysis was done to the weekly mean of daily peakMET values both in absolute and relative terms.

**Results:**

The CRF correlated significantly with absolute peakMET (r = 0.500) and relative peakMET (r=-0.456). In absolute terms, the highest CRF third had the highest peakMET value for men (6.5±1.8; 6.0±1.5; 5.2±1.2) and women (6.3±1.4; 5.8±1.1; 5.2±1.0). In relative terms, the highest CRF third utilized least of their aerobic capacity (men: 53±14%; 56±13%; 61±15%) and (women: 58±12%; 60±12%; 70±16%).

**Conclusion:**

One minute of the day can provide valuable information about the individual’s CRF and the level of effort required during PA. Higher measured absolute peakMET may indicate higher CRF, as more fit individuals can sustain higher levels of PA. When an individual’s CRF is taken into account, individuals with lower CRF have to utilize more of their aerobic capacity. Thus, high CRF provides a possibility to operate at higher intensity levels and protects from strenuous PA during daily routines.

